# Adapting floating offshore wind-hydrogen systems for emerging markets: the case of the Ica region, Peru

**DOI:** 10.1007/s40868-026-00215-1

**Published:** 2026-02-18

**Authors:** Claudio A. Rodríguez, Maurizio Collu, Feargal Brennan

**Affiliations:** University of Strathclyde, 100 Montrose St, Glasgow, G4 0LZ UK

**Keywords:** Offshore wind turbine, Energy transition, Green hydrogen

## Abstract

The transition to sustainable energy systems is essential for reducing the carbon footprint of maritime and port infrastructure, particularly along South America’s Pacific coast. This paper investigates the design of a floating platform for stand-alone offshore wind-powered hydrogen production, focusing on the central coast of Peru, which holds exceptional offshore wind potential. The system integrates hydrogen production facilities with the IEA 15-MW reference floating offshore wind turbine mounted on a semi-submersible platform, emphasizing key design aspects from the perspective of motion dynamics. Stability and site-specific hydrodynamic analyses are conducted under representative sea states and wind conditions of the Ica offshore region to evaluate the accelerations imposed on hydrogen production equipment. Results highlight the benign yet energetic environment of Ica, which offers favourable wind consistency, moderate waves, and reduced extremes compared to benchmark North Atlantic sites. These findings confirm the technical feasibility of floating wind–hydrogen integration in Peru, demonstrating that platform responses remain well within recommended design criteria. By coupling renewable power generation with hydrogen production at sea, the study demonstrates a pathway to decarbonize port operations and strengthen the sustainability and resilience of maritime infrastructure in Peru and the wider Pacific region.

## Introduction

The Pacific coast of South America is undergoing rapid transformation in both its energy and maritime sectors. In Peru, large-scale infrastructure investments—such as the new multipurpose Port of Chancay, alongside existing hubs like Pisco, Callao, and the LNG terminal at Pampa Melchorita—reflect the country’s strategy to modernize logistics networks and expand international trade. At the same time, Peru has committed to achieving carbon neutrality by 2050 [1], a target that demands deep decarbonisation across transport, port operations, and heavy industry, all of which remain heavily reliant on fossil fuels.

Among the most promising pathways for this transition is floating offshore wind-powered hydrogen production. This approach enables direct offshore conversion of wind-generated electricity into hydrogen, a storable and transportable zero-carbon energy carrier. It is particularly suited to deep-water environments such as the Ica coast, identified by the World Bank [2] and the Global Wind Atlas [3] as Peru’s most promising offshore wind resource, with average wind speeds in the IEC Class I/II range and bathymetric conditions favourable to floating platforms.

This paper examines the adaptation of a floating wind–hydrogen system (FWHS), originally developed under the Ocean REFuel programme [4] in the UK, to a representative site near Ica, Peru. Using high-resolution ERA5 [5] wind and wave reanalysis data (2004–2024), the study evaluates wind resource availability, expected capacity factors for the IEA 15-MW reference turbine, operational windows, and the hydrodynamic performance of a semisubmersible hydrogen platform under local metocean conditions. The proposed system integrates modular hydrogen production equipment directly onto the floating wind platform, eliminating the need for long-distance submarine power export cables.

Beyond technical feasibility, the project is framed as a catalyst for sustainable maritime development. The platform could supply green hydrogen to ports for shore-side power, bunkering, and industrial applications, directly supporting the decarbonisation of port infrastructure—an urgent priority as Peru’s port network expands. The proximity to Pampa Melchorita further opens opportunities for hydrogen or ammonia export by leveraging Peru’s established LNG handling, storage, and shipping capacity.

International precedents reinforce this vision: the Port of Rotterdam is repurposing LNG terminals for hydrogen trade; Japan is adapting LNG facilities for liquefied hydrogen and ammonia; and Chile is converting coastal LNG hubs into export-oriented hydrogen corridors. These examples demonstrate that floating hydrogen systems are moving rapidly from concept to implementation as part of integrated energy strategies. With abundant wind resources and emerging port infrastructure, Peru is well positioned to lead South America’s offshore hydrogen transition.

By tailoring this solution to Peru’s coastal environment and infrastructure landscape, this study seeks to bridge the gap between European innovations and Latin American needs. The findings provide timely insights for policymakers, port authorities, and energy stakeholders developing integrated, scalable pathways toward national and regional net-zero targets.

The remainder of this paper is structured as follows. Section 2 reviews the current status of renewable energy in Peru and identifies the offshore wind potential of the Ica region. Section 3 describes the methodology and datasets used for wind and wave characterization, together with extreme value analyses. Section 4 presents the adaptation of the floating wind–hydrogen system to Ica, including stability, hydrodynamic performance, and hydrogen production assessments. Finally, Sect. 5 discusses the broader implications for Peru’s maritime energy transition and concludes with perspectives for future research and deployment.

## Offshore renewable energy in Peru

### Onshore wind experience

Since Peru’s first onshore wind farm came online in 2012, cumulative installed capacity has reached 668 MW, representing about 3% of the country’s estimated 20.5 GW onshore technical potential. Practical experience has underscored both the opportunities and limitations of onshore projects [6]. On the positive side, onshore wind farms have demonstrated a relatively low environmental footprint in non-protected areas, facilitated job creation during construction phases, and enabled straightforward operation, maintenance, and workforce training. At the same time, challenges remain, particularly the intermittency of the wind resource, its lack of inherent dispatchability, and potential site conflicts in environmentally sensitive or culturally protected areas.

Despite these limitations, onshore wind development has built valuable technical and institutional capacity in Peru. It has also fostered community acceptance and proven that wind power can be a viable complement to the nation’s predominantly hydro-thermal energy mix.

### Offshore wind hotspots in South America

Preliminary offshore resource assessments using the Global Wind Atlas v3.4 [3] at 150 m hub height (typical of IEC Class I turbines such as the IEA 15-MW reference [7]) reveal only a handful of high-potential marine “sweet spots” across Latin America. These include the northern Caribbean coast (Barranquilla, Colombia to Maracaibo, Venezuela), the southern Pacific coast of Chile (from La Serena southwards), the southern Atlantic coast (South of Brazil, Uruguay, and Argentina), and the offshore region of Ica, Peru.

**Fig. 1:**
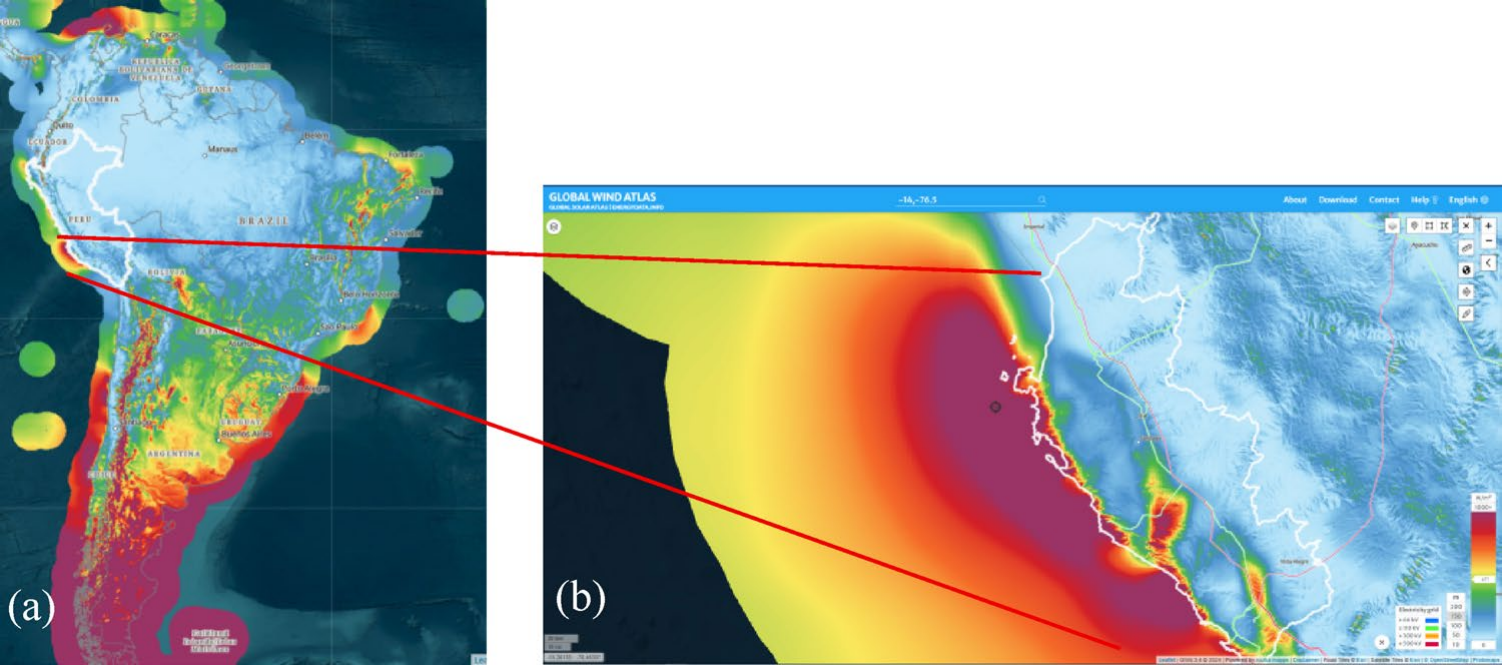
**a** Mean offshore wind power density at 150 m above mean sea level for South America [3]. Only four coastal hotspots exceed 1000 W/m^2^. **b** Zoom-in of the Ica offshore region. The black dot indicates the point used for site characterization

Figure 1a illustrates the mean offshore wind power density across South America, highlighting these few exceptional zones. Among them, the Ica offshore region stands out as the only high-potential area in the central part of the continent, offering power densities comparable to higher-latitude sites but without exposure to extreme sea states. A closer view in Fig. 1b shows that this offshore wind corridor spans more than 250 km of coastline and extends approximately 50 km offshore—an area larger than the horizontal footprint of any single ScotWind lease zone.

This vast, energetic region combines several attributes that make it particularly promising for floating offshore wind and integrated hydrogen production: (i) mean offshore power densities exceeding 1000 W/m^2^ at 150 m, (ii) a relatively moderate wave climate compared to southern Chile or Brazil, and (iii) proximity to deep waters (> 100 m), where floating rather than fixed-bottom platforms are technically optimal.

### Regulatory & infrastructure context

From a regulatory perspective, the Peru’s Renewable Energy Regulatory Index (RISE) score of ~ 50/100 in 2020 signals both progress and gaps [2]. While policy support for renewable energy is growing, offshore wind still lacks a dedicated leasing framework, tailored environmental impact assessment (EIA) guidelines for floating structures, and standards for grid interconnection or export-grade hydrogen certification.

At the same time, major infrastructure investments are reshaping the coastal corridor between Pisco and Chancay. The new multipurpose Port of Chancay, ongoing upgrades at the Pampa Melchorita LNG terminal, and the concentration of large-scale mining and agro-industrial clusters all represent potential off-takers and logistical anchors for early offshore hydrogen projects. Together, these assets create a favourable setting for pilot deployments. With coordinated permitting, environmental safeguards, and strategic infrastructure investment, Peru could accelerate the integration of offshore wind and hydrogen production, leveraging both its regulatory momentum and existing industrial synergies [8].

Environmental viability is a critical consideration for offshore development in Peru. Several marine-coastal areas in the Ica region fall under national or regional protection schemes, including marine reserves and areas of high biodiversity. While the present study focuses on the technical and resource characterization of offshore hydrogen platforms, any future deployment would require detailed Environmental Impact Assessments (EIAs) in compliance with Peruvian legislation and international best practices. This would involve careful site selection to avoid overlap with protected zones, assessment of potential effects on marine habitats, and integration of mitigation measures. In this sense, the favourable metocean and infrastructural conditions identified here should be viewed as a starting point, with environmental licensing and marine spatial planning processes serving as decisive next steps.

## Methodology and data

### Wind resource

To characterize the offshore wind climate of the Ica region, ERA5 reanalysis data from the Copernicus Climate Data Store [5] was retrieved for the period January 2004 -December 2024 at a representative location within the offshore hotspot (latitude − 14.0°, longitude − 76.5°), approximately 200 m water depth. Wind speeds were extracted at 150 m above mean sea level—corresponding to the hub height of the IEA 15-MW reference wind turbine. For comparison, the NE8 site [9] in northeast Scotland (latitude 58.5°, longitude − 1.0°, approx. 100 m water depth) was also analysed using ERA5 data (2002–2021) as a benchmark North Sea environment.

**Fig. 2:**
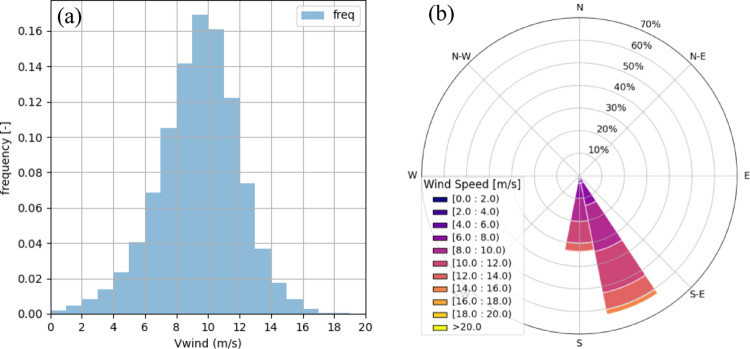
**a** Histogram of wind speed at 150 m above mean sea level for the Ica offshore site (2004–2024). **b** Wind speed rose for Ica offshore site at 150 m height

Figure 2a shows the wind speed histogram for Ica. The distribution is notably consistent, with peaks at 9–10 m/s (17% occurrence) and 10–11 m/s (16%). Wind speeds rarely exceed 20 m/s and remain well below the turbine’s 25 m/s cut-off. In contrast, NE8 displays a broader spread with values up to 35 m/s and only ~ 7.5% occurrence per bin in the modal ranges. This contrast suggests a more stable and predictable wind regime in Ica, potentially reducing control-system demands, particularly in yaw and pitch regulation.

**Wind direction analysis.** The wind rose in Fig. 2b further illustrates the highly directional character of the Ica resource: more than 90% of winds originate from two adjacent 22.5° sectors centred at 157.5° and 180° (SSE to S). At NE8, by contrast, winds are distributed across multiple sectors, requiring frequent nacelle yaw adjustments. The directional stability at Ica is therefore expected to reduce yaw-induced wear and improve system reliability.

**Wind power distribution and capacity factor.** Using the IEA 15-MW turbine power curve (cut-in: 3 m/s; rated: ~ 10.5 m/s; cut-off: 25 m/s), the wind power distribution reveals:

- Full-rated power (~ 15 MW) reached in ~ 32.5% of the dataset.
- Near-uniform output between 4–14 MW (~ 5% per 1-MW bin).
- Downtime due to insufficient wind (< 3 m/s) accounts for only 1.48%.
- No cut-off events (> 25 m/s) were recorded.

At NE8, full-rated power occurs more frequently (47.1%), but total downtime reaches 6.36% due to both low and extreme winds. NE8 also records operation in the 0–2 MW range ~ 13% of the time, highlighting more frequent underperformance intervals. Despite operating fewer hours at full capacity, Ica achieves a higher average capacity factor (66.8%) compared to NE8 (64.6%), reflecting the steadier mid-to-high wind resource.

**Operational and downtime windows.** To assess wind power availability continuity, operational and downtime windows were computed. An operational window is defined as a continuous period where P_gen_ > 0 MW, and a downtime window is a continuous period where the wind speed is either below cut-in (< 3 m/s) or above cut-off (> 25 m/s), i.e., the wind turbine is not generating energy.

**Fig. 3:**
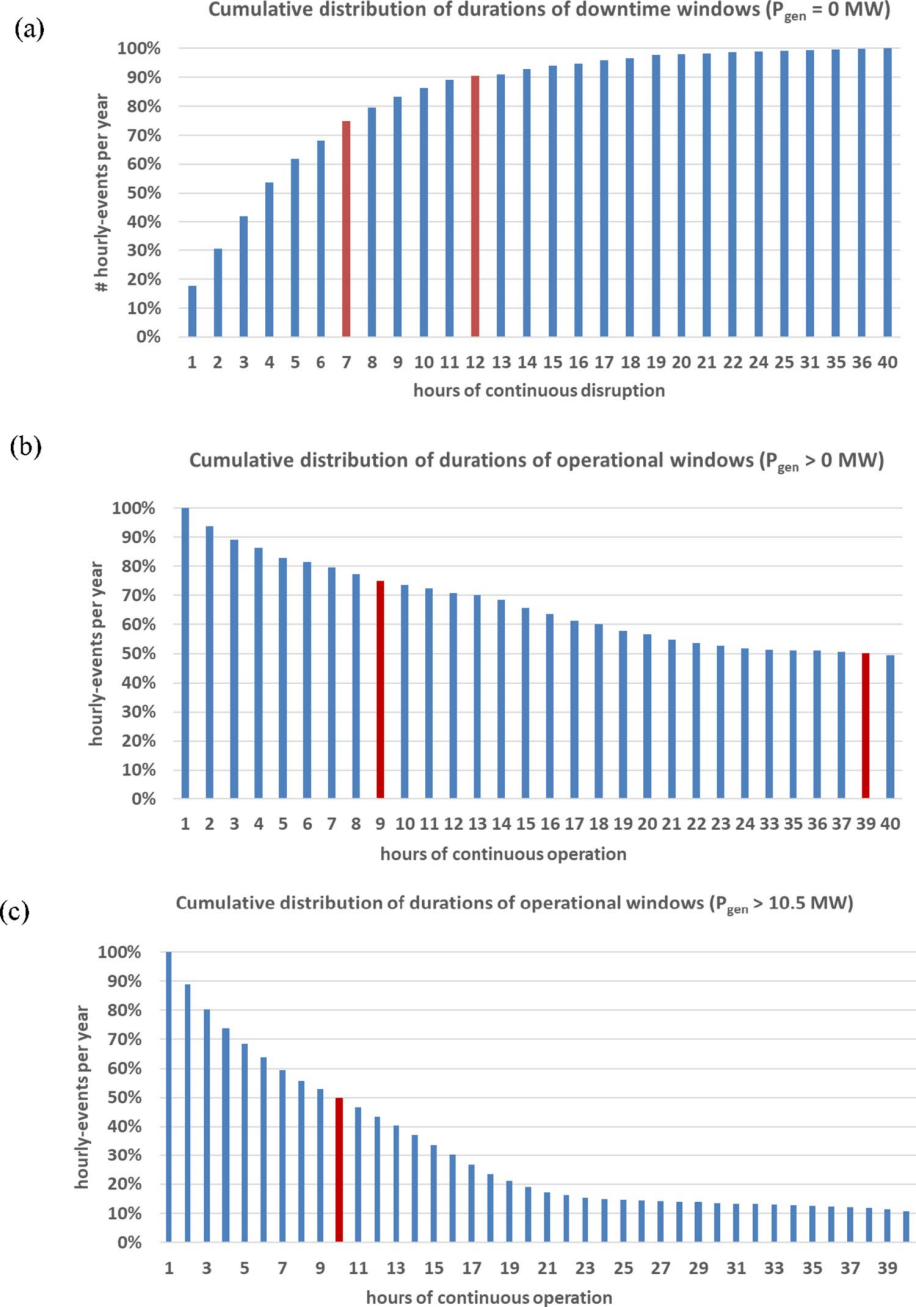
Cumulative distribution of window durations at the Ica offshore site: **a** downtime, **b** operational, **c** high power output events (> 10.5 MW)

For Ica, downtime averages 22 events/year with mean duration ~ 6 h (max: 40 h). Seventy-five percent of events last ≤ 7 h, and 90% ≤ 12 h (Fig. 3a). Operational windows are comparatively long: 50% exceed 39 h, and 75% exceed 9 h (Fig. 3b). Roughly half of the high-output events (P_gen_ > 10.5 MW) last longer than 10 h (Fig. 3c).

At NE8, downtime averages 98 events/year, also ~ 6 h mean duration, but with a maximum of 57 h. Although high-output windows are longer (50% > 16 h, 75% > 160 h), interruptions are significantly more frequent. Overall, Ica offers greater regularity and predictability, features advantageous for electrolyser scheduling and hydrogen storage design.

### Wave climate

The wave climate was evaluated using ERA5 hindcast data for the same 2004–2024 period. Significant wave height (Hs), peak wave period (Tp), and direction were analysed to quantify environmental constraints and implications for platform motions.

**Fig. 4:**
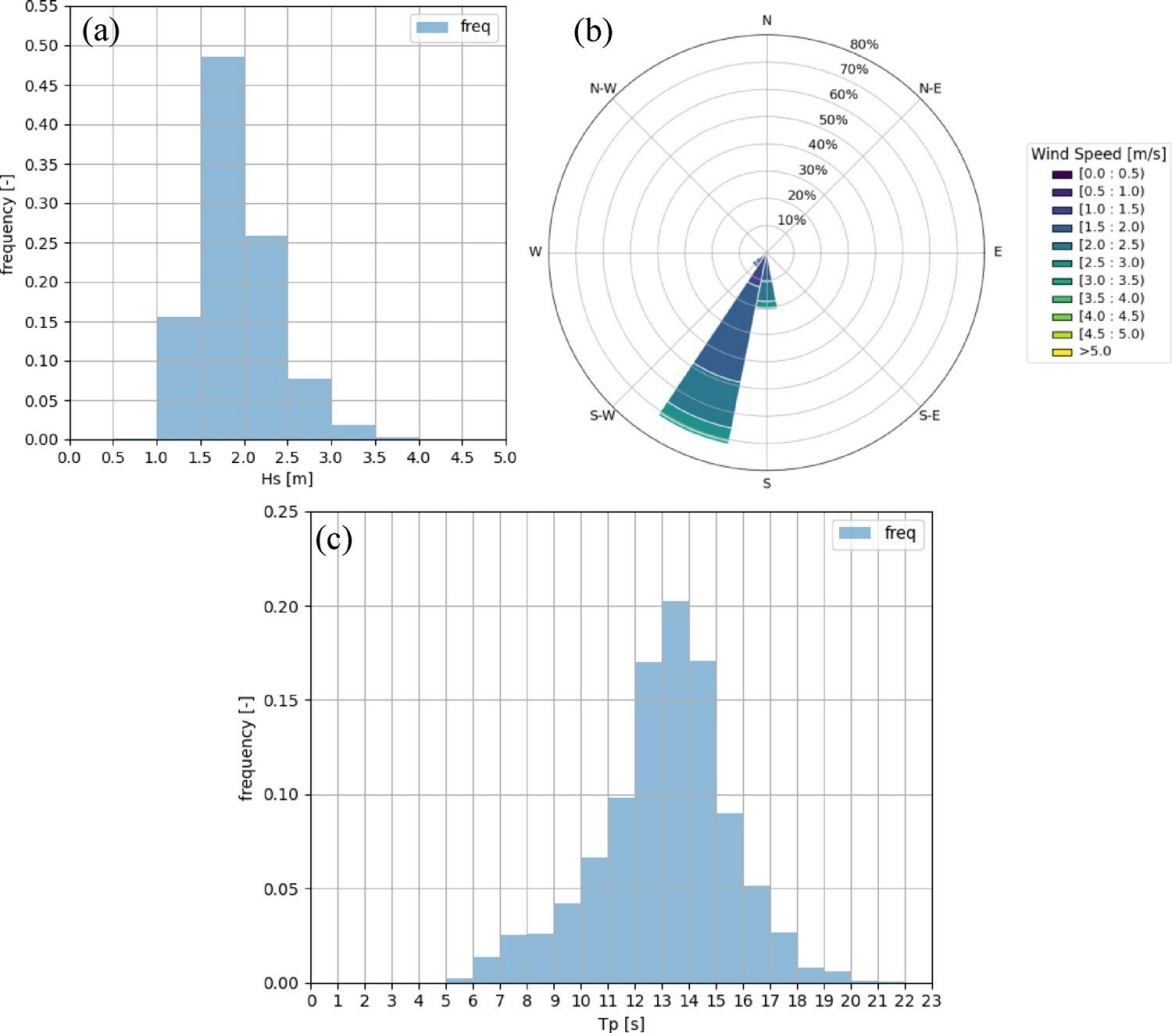
**a** Histogram of significant wave heights, **b** Wave rose, **c** Histogram of peak periods, for the Ica offshore site (2004–2024)

**Wave height.** The histogram of Hs (Fig. 4a) shows that ~ 48% of waves lie in the 1.5–2.0 m range. Maximum Hs over the 20-year record was 4.86 m, and ~ 90% of states are below 2.5 m. This confirms a benign wave environment compared with the North Atlantic. At NE8, ~ 60% of waves are below 2.0 m, but extremes reach 9.38 m, with 10% exceeding 3.5 m.

**Wave direction and period.** Directional analysis (Fig. 4b) shows > 70% of waves from SSW, with another 10% from the south. This aligns closely with the wind rose, indicating strong metocean consistency and simplifying station keeping strategies. Most peak wave periods fall within 12–15 s (Fig. 4c), characteristic of swell-dominated conditions. NE8, conversely, is dominated by shorter waves (5–10 s in > 70% of cases), leading to steeper seas with stronger nonlinear loading.

While the wave climate results presented here are based on ERA5 reanalysis data, it is recognised that such global products may not fully capture nearshore dynamics along the Peruvian coast, particularly processes driven by local bathymetry, swell refraction, and upwelling-related wind–wave interactions. Previous studies have shown that ERA5 tends to smooth extreme events and underrepresent the spatial variability of significant wave height (Hs) in coastal regions. For Peru, national agencies such as IMARPE (Instituto del Mar del Perú) maintain in-situ buoy networks, tide gauges, and regional models that can provide higher-fidelity datasets for validation. Future work will therefore include the integration of IMARPE measurements and regional model outputs to refine the metocean characterisation of the Ica offshore hotspot. This will enable more accurate design load assessments and ensure consistency with regulatory expectations and local environmental baselines.

### Extreme value analysis

A preliminary extreme value analysis (EVA) was carried out for both wind speed and Hs using the Generalized Extreme Value (GEV) distribution. A dual-method approach was employed to ensure robustness. First, extreme values were identified using a Peaks-Over-Threshold (POT) method with a 3-day declustering window. These peaks were fitted to a Generalized Pareto (GP) distribution. Second, the classical Annual Maxima Series (AMS) was extracted and fitted to a Generalized Extreme Value (GEV) distribution. For both methods, a non-parametric bootstrap procedure with 1,000 iterations was used to estimate the 50-year return values and their 95% confidence intervals, providing a measure of the fitting uncertainty. The final values presented in Table 1 are the median estimates from the GEV distribution fitted to the annual maxima. Weibull fits were considered but discarded due to poor tail extrapolation for waves. GEV provided consistent estimates, showing substantially lower extremes for Ica compared to NE8. Table 1 summarises the 1-, 10-, and 50-year return values for wind speed at 10 m height (above mean water level) and significant wave height.

**Table 1:** Comparison of extreme metocean conditions for the Ica and NE8 offshore sites

Return Period	Wind speed [m/s]		Sig. Wave height [m]	
	Ica	NE8	Ica	NE8
1-year	13.8	23.4	4.2	8.8
10-year	14.0	26.1	4.3	9.7
50-year	14.9	29.0	4.9	11.4

To estimate the concurrent extreme wind speed at hub height (150 m), a site-specific wind shear exponent (α) was derived. This exponent was calculated based on the 50-year return wind speeds estimated at 10 m and 100 m heights from the same ERA5 dataset, under the assumption that the extreme meteorological event drives correlated extremes at different heights within the atmospheric boundary layer. The derived value was α = 0.105.

**Wind.** At Ica, the 50-year return wind speed is 14.9 m/s at 10 m MSL (equivalent to ~ 20.9 m/s at hub height). This remains well below the 25 m/s cut-off of the IEA 15-MW turbine, suggesting uninterrupted operation during rare events. At NE8, the 1-year return wind speed (23.4 m/s at 10 m) already approaches the cut-off, while the 50-year return (29.0 m/s) exceeds it, increasing curtailment risk.

**Waves.** The 50-year Hs at Ica is 4.9 m, less than half the NE8 value of 11.4 m. This translates to substantially lower hydrodynamic loading, reducing substructure and mooring demands. The milder environment also limits accelerations and sloshing loads on hydrogen production systems, enhancing operational reliability.

Together, these results underline Ica’s unique combination of strong yet stable winds, benign waves, and lower extremes. This profile improves not only the feasibility of floating wind deployment but also the integration of motion-sensitive hydrogen production equipment.

## Platform and system design adaptation for the Ica offshore region

The proposed Floating Wind–Hydrogen System (FWHS) for deployment off the coast of Ica, Peru, is derived from the configuration previously developed and validated for the NE8 lease site in Scotland. It integrates the open-source IEA 15-MW reference wind turbine [7] with the UMaine VolturnUS-S three-column semi-submersible platform [10] and an onboard, decentralised hydrogen production plant. While the configuration strategy follows the earlier study [11], adaptations are introduced to account for the specific metocean environment of the Ica region.

### System configuration overview

**Fig. 5:**
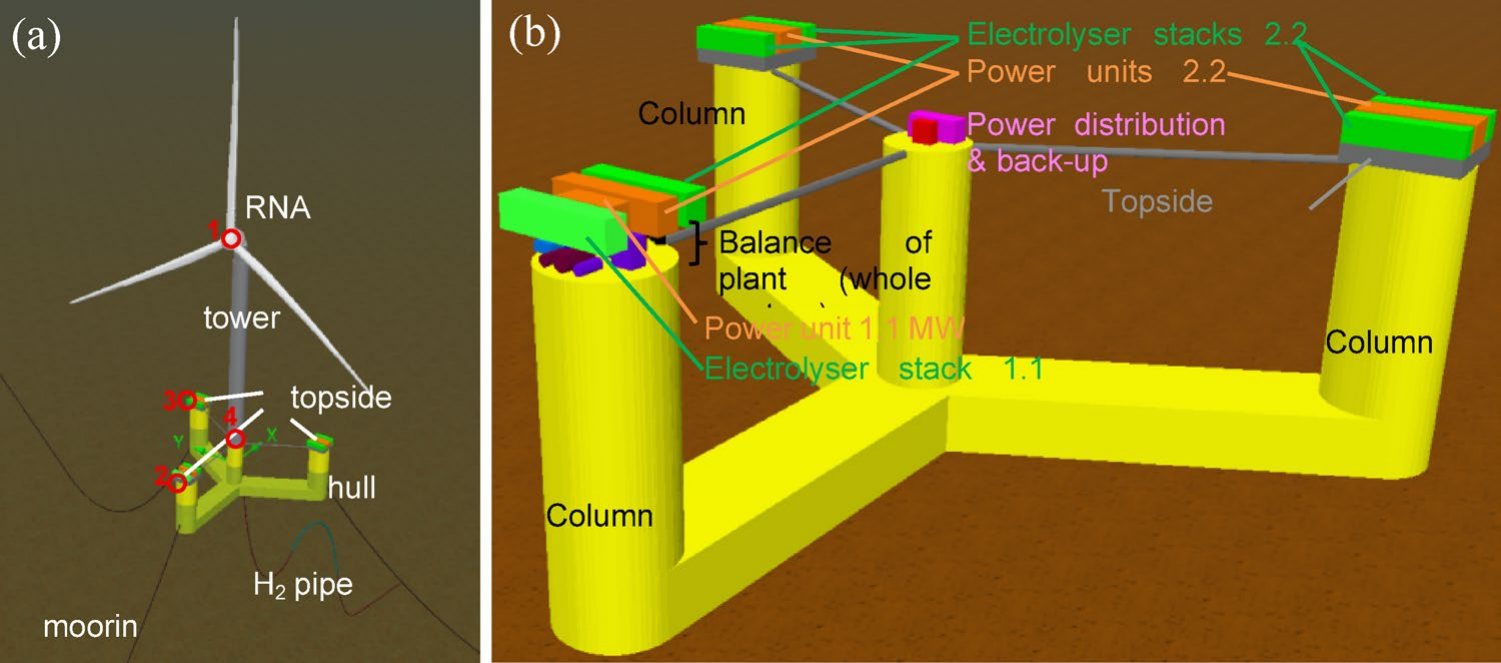
**a** Floating Offshore Wind-Hydrogen layout. The red dots mark critical locations considered for the hydrodynamic assessment of the system. **b** Hydrogen production equipment

The IEA 15-MW wind turbine is mounted on the UMaine VolturnUS-S platform using the tower designed for the reference FOWT. The turbine operates at a rated wind speed of ~ 10.6 m/s, with cut-in and cut-off thresholds of 3 m/s and 25 m/s, respectively. The semi-submersible provides hydrostatic stability, mooring interfaces, and space for hydrogen production facilities. Electrolysis is conducted onboard via a modular, containerized PEM system powered directly by the turbine. The electrolyser units are distributed across the topside of the three columns for blast isolation, while mooring relies on a conventional three-line catenary system. Hydrogen export is managed through a flexible riser in a lazy-S configuration (Fig. 5a).

The entire configuration—including turbine, platform, mooring, and hydrogen plant—is retained as per the reference case for NE8 to enable direct performance comparisons and ensure compatibility with validated design frameworks. Optimisation for local conditions is anticipated in future phases.

**Hydrogen system main characteristics.** The hydrogen facility comprises six PEM electrolyser units, totalling 12.1 MW of capacity: five Nel® MC500 modules (2.2 MW each) and one MC250 (1.1 MW). This configuration balances compatibility with the wind power profile and deck space limitations.

In addition to the electrolysis stacks and power handling units (rectifiers/transformers), the hydrogen production system includes the balance of plant (BoP), which encompasses all auxiliary systems required for safe and continuous operation of the hydrogen facility. In this context, BoP refers to the water intake and treatment units, power distribution and conversion systems, cooling systems, backup power supply, chemical dosing and venting systems, and all associated safety, and control equipment. The main characteristics of the hydrogen production system, including electrolyser configuration, BoP equipment, and performance parameters, are summarised in Table 2.

**Table 2:** Main characteristics of the hydrogen production system

Component	Model/Description	# Units	Capacity/Spec	Footprint (L × D × H)	Weight per unit (t)
Electrolyzer Modules	Nel MC500 (PEM)	5	492 Nm^3^/h Hz per unit (~ 44 kg/h)	12.2 × 2.5 × 3.0 m	18.6 (process) /24.0 (power)
	Nel MC250 (PEM)	1	246 Nm^3^/h Hz (~ 22 kg/h)	12.2 × 2.5 × 3.0 m /6.1 × 2.5 × 2.6 m	17.3/18.0
Water Treatment System	Desalination + deionization	1	~ 3.55 m^3^/h purified water	1.8 × 0.8 × 1.0 m	1.0
Seawater Lift System	Pumps + strainers MV/LV	2	416 m^3^/h total	2.0 × 1.2 × 1.0 m	0.3
Power Distribution	Transformer (Tower Base)	1	1500 kVA	2.4 × 1.5 × 2.5 m	3.9
Backup Power System	Back-start battery	1	2 MWh	6.1 × 2.4 × 2.6 m	28.0
Auxiliary Systems	Instrument air, safety, control	1	760 Nm^3^/h air	2.3 × 1.8 × 2.7 m	2.3

The electrolysers are mounted on dedicated support structures on each platform column, adding ~ 465 t to the topside mass. Including equipment, the hydrogen plant contributes ~ 765 t to the system. The nominal hydrogen production rate is ~ 243 kg/h at 30 bar, with freshwater consumption of ~ 2.5 m^3^/h. Electrolysers can operate at loads as low as 5% of nominal, corresponding to ~ 56 kW.

### Hydrostatic stability

The addition of ~ 765 t of hydrogen systems—including support structures—raised the platform’s vertical centre of gravity (VCG) by 1.36 m. To maintain the original draft and trim of the reference FOWT, seawater ballast adjustments were introduced. This preserves total system mass while limiting changes to pitch and roll inertia (< 6%).

**Fig. 6:**
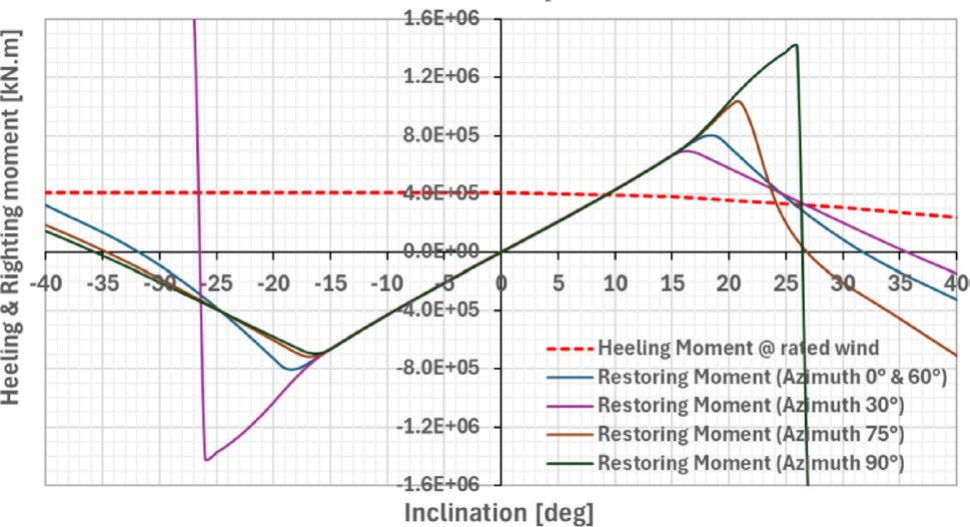
Static heeling and stability restoring moments for different azimuths

Quasi-static stability was assessed according to DNV-ST-0119 [12] (Fig. 6). The applied heeling moment corresponds to rated wind speed conditions (~ 10.5 m/s). For azimuths between 0° and 60°, the platform does not fully satisfy the minimum safety factor (γ_stab_ ≥ 1.30), yielding values of 1.20 (0°/60°), 1.13 (15°/45°), and 1.11 (30°). For the assessed headings, the DNV criterion is met for azimuths of 75° and 90°. This indicates that the platform’s stability is direction-dependent and may benefit from optimising platform heading to align these stable orientations with the prevailing wind-wave directions at Ica. Dynamic simulations should also support potential further refinement.

### Hydrodynamic performance

**Frequency domain analysis.** A frequency-domain hydrodynamic analysis was performed without external damping, providing a conservative, site-independent baseline. Four critical locations were selected for monitoring (Table 3 and Fig. 5a), covering the RNA hub, electrolyser positions, and hydrogen export pipe.

**Table 3:** Coordinates of critical locations for hydrodynamic assessment

Id	Critical locations	x [m-TC]	y [m-TC]	z [m-MWL]
1	RNA hub	− 13.644	0.000	150.170
2	Electrolyser col. 1	− 58.000	6.250	21.000
3	Electrolyser col. 2	32.125	51.067	21.000
4	H_2_ pipe connection	6.000	6.000	15.000

**Fig. 7:**
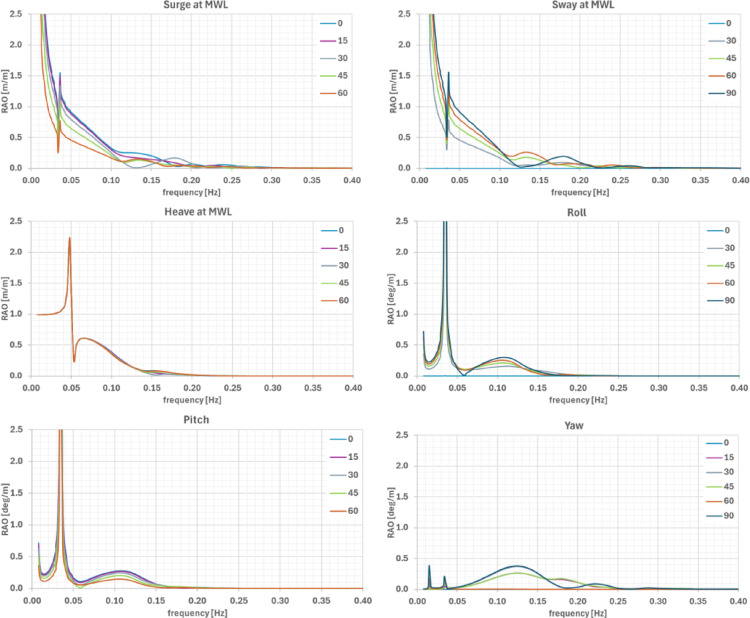
Six-degree-of-freedom motions RAOs in surge, sway, heave, roll, pitch and yaw, for different wave headings. Translational motions are defined at the platform’s origin at mean-water level (MWL)

Eigenvalue analysis identified natural periods (Table 4) closely aligned with peaks in the RAOs curves. As shown in Fig. 7, surge and sway responses were dominated by natural frequencies, with secondary peaks at ~ 0.133 Hz and ~ 0.182 Hz. Heave exhibited a secondary peak of ~ 0.60 m/m at 0.067 Hz, while roll and pitch displayed secondary peaks at 0.111 Hz (0.15–0.30 m/m, depending on heading). Yaw showed peaks near 0.125 Hz (0.30–0.40 deg/m) and at ~ 0.182 or 0.222 Hz.

**Table 4:** Eigen-frequencies from frequency-domain hydrodynamic analyses

Mode	Surge	Sway	Heave	Roll	Pitch	Yaw
Freq. [Hz]	0.008	0.008	0.051	0.035	0.035	0.016
Period [s]	125.3	125.3	19.8	28.4	28.5	61.2

**Fig. 8:**
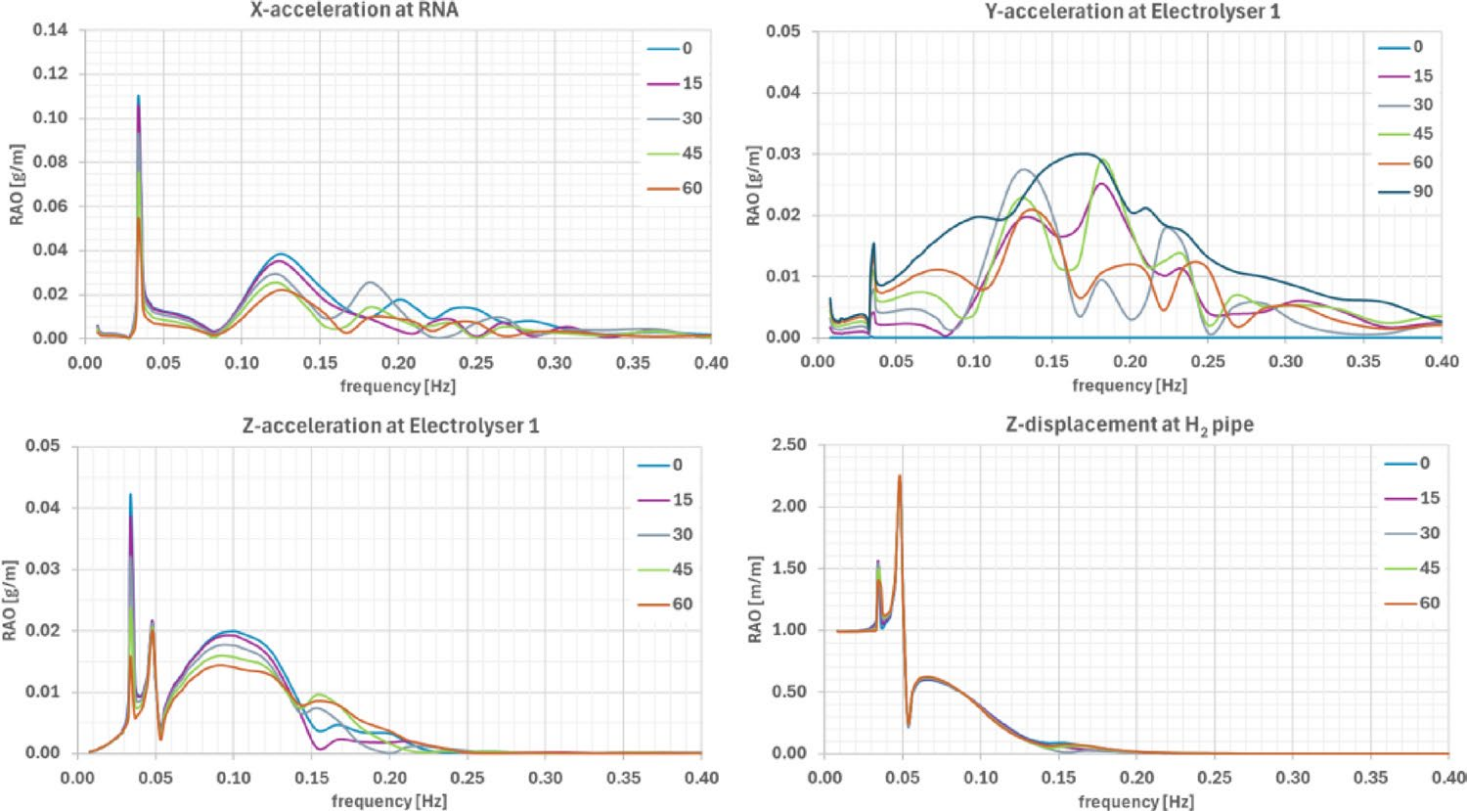
RAOs of X- Y- and Z-acceleration at RNA and electrolyser locations and, of the Z-displacement at H_2_ pipe connector, for different wave headings

Peak accelerations confirm the frequency-dependent amplification. As shown in Fig. 8, at the RNA hub, the largest accelerations (~ 0.11 g/m) occur in the horizontal components near the pitch/roll natural frequencies (~ 0.035 Hz), with a secondary peak observed at ~ 0.125 Hz. At the electrolyser locations, the dominant acceleration component is vertical (~ 0.045 g/m), primarily associated to the pitch natural frequency, with smaller secondary wide humps at higher frequencies. For the horizontal components, although the peak amplitudes are not as pronounced as in the vertical component, multiple peaks appear distributed across a relatively wide frequency range. Displacements at the H₂ pipe connection are larger at very low frequencies—likely associated to surge/sway (< 0.013 Hz) but exhibit notable contributions also at heave and pitch natural frequencies.

These site-independent results provide a conservative indication of the system’s motion and acceleration characteristics and identify frequencies where dynamic amplification may occur. Following these RAOs will be combined with Ica’s sea states to quantify platform’s responses at operational and survival conditions.

*Spectral responses for Ica’s sea states.* Two representative design load cases were selected to evaluate the hydrodynamic performance of the Floating Wind–Hydrogen System (FWHS) at Ica:

- **Operational condition:** Defined as the sea state associated with the mean wind speed at hub height equal to the turbine’s rated wind speed (10.6 m/s), determined via regression of hindcast *H*_s_ and wind speed data.
- **Survival condition:** Based on the 50-year *H*_s_ and associated *T*_p_ determined from IEC 61400-3-2:2025 [13], assuming swell-dominated waves (consistent with observed metocean data).

**Fig. 9:**
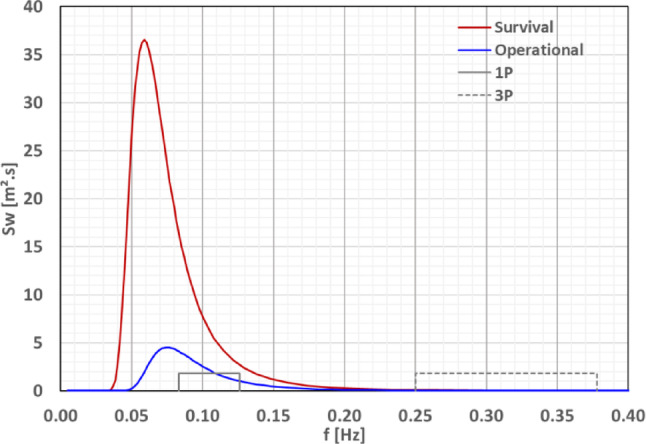
Representative sea sates (operational and survival) with IEA-15 MW rotor excitation frequencies

The significant wave heights and peak periods for both conditions are given in Table 5. For each, a JONSWAP spectrum was defined with the peak enhancement factor γ computed from DNV-RP-C205 [14]. The resulting spectra are shown in Fig. 9. These spectra were combined with the platform RAOs to generate displacement and acceleration response spectra at the critical locations listed in Table 3 for headings from 0° to 180°. From these, significant response amplitudes and associated periods were obtained. A total of 468 spectra (2 sea states × 18 motion parameters × 13 headings) were analysed, where the 18 parameters correspond to 6 DOFs (surge, sway, heave, roll, pitch and yaw) at the platform’s origin plus 3 motion components at the four critical locations (displacement at the connector of the H_2_ export pipe, acceleration at the electrolysers on columns 1 and 2, and acceleration at the RNA).

**Table 5:** Representative design loading conditions (DLC) for the FWHS for the Ica region

DLC	WT Mode	Wind		Sea state		
		Type	V_hub_ [m/s]	Hs [m]	Tp [s]	γ [–]
1.3	Power Production (operational)	ETM	10.59	1.95	13.3	1.00
1.6	Power Production (operational)	NTM	10.59	4.90	17.0	1.00
6.1	Parked (survival)	EWM	20.86	4.90	17.0	1.00

**Fig. 10:**
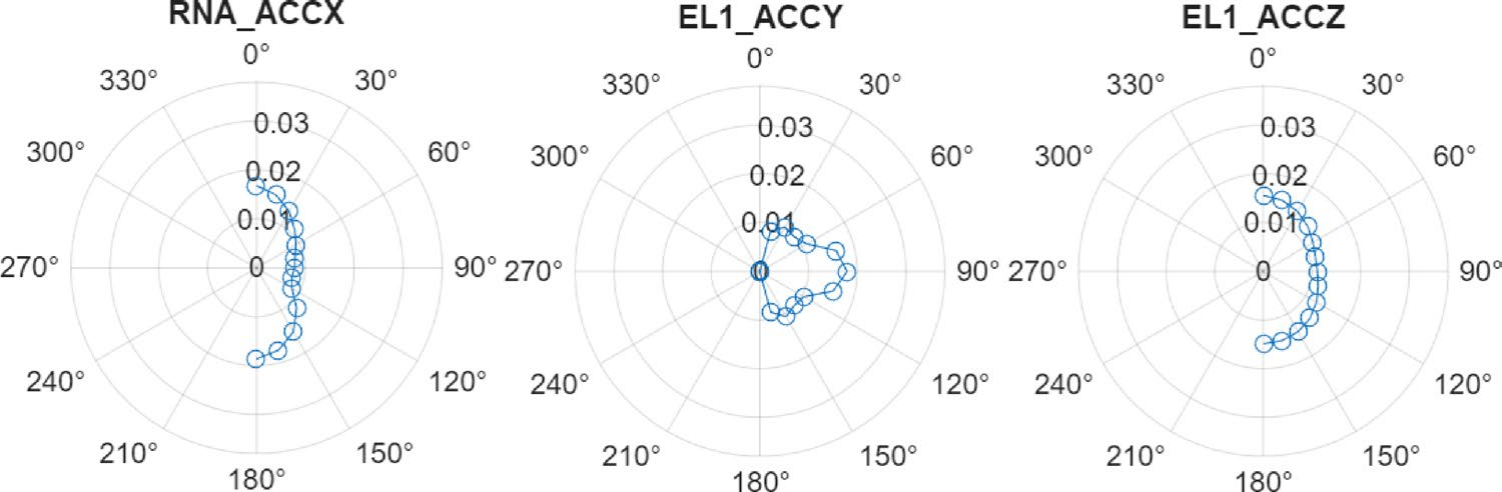
Significant acceleration components for the most critical locations at the platform under operational sea conditions: frequency-domain approach

**Fig. 11:**
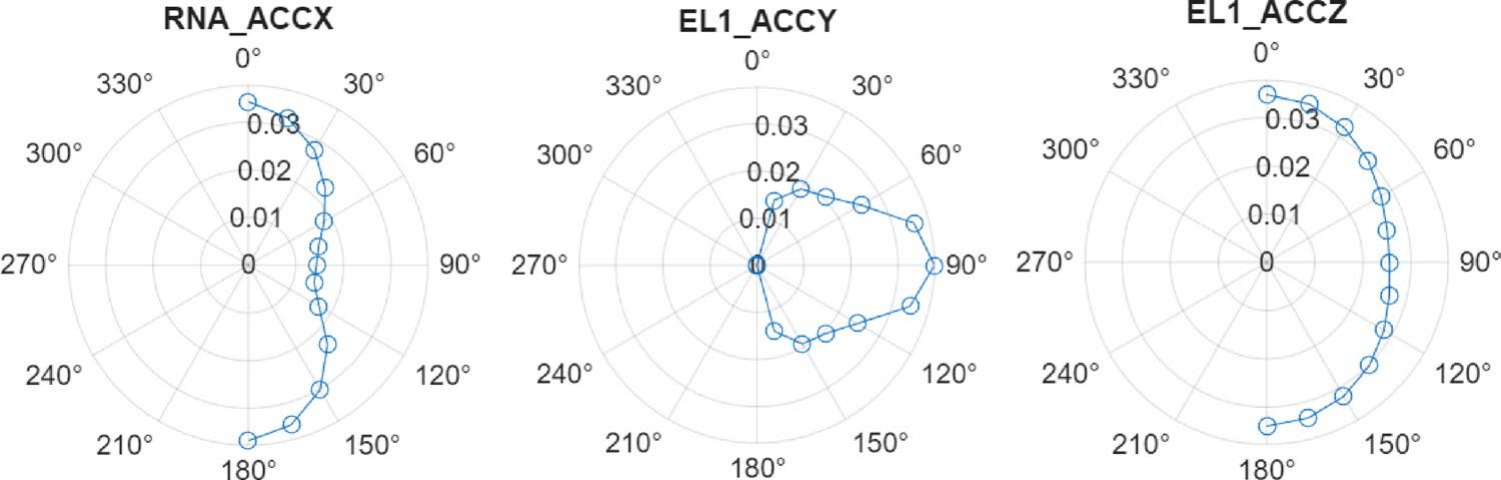
Significant acceleration components for the most critical locations at the platform under survival sea conditions: frequency-domain approach

Polar plots of maximum significant accelerations for the operational and survival conditions are presented in Fig. 10 and Fig. 11, respectively. Key findings include:

- **RNA hub:** Max acceleration in x-direction at 180°—0.037 g (survival).
- **Electrolyser at Column 1:** Highest response overall, 0.038 g (survival) and 0.016 g (operational) in y-direction at 90°.
- **Electrolyser at Column 2/3:** 0.034 g in y/z directions, with incidence dependence between and 15°–105°. Operational values are ~ 0.013–0.015 g.

All acceleration levels remain well below DNV-RP-C103 [15] for column-stabilised units and far below RNA acceleration tolerances in DNV-RP-0286 [16] (0.3 g operational, 0.6 g survival). Since only radiation damping was included, actual values are expected to be even lower.

**Fig. 12:**
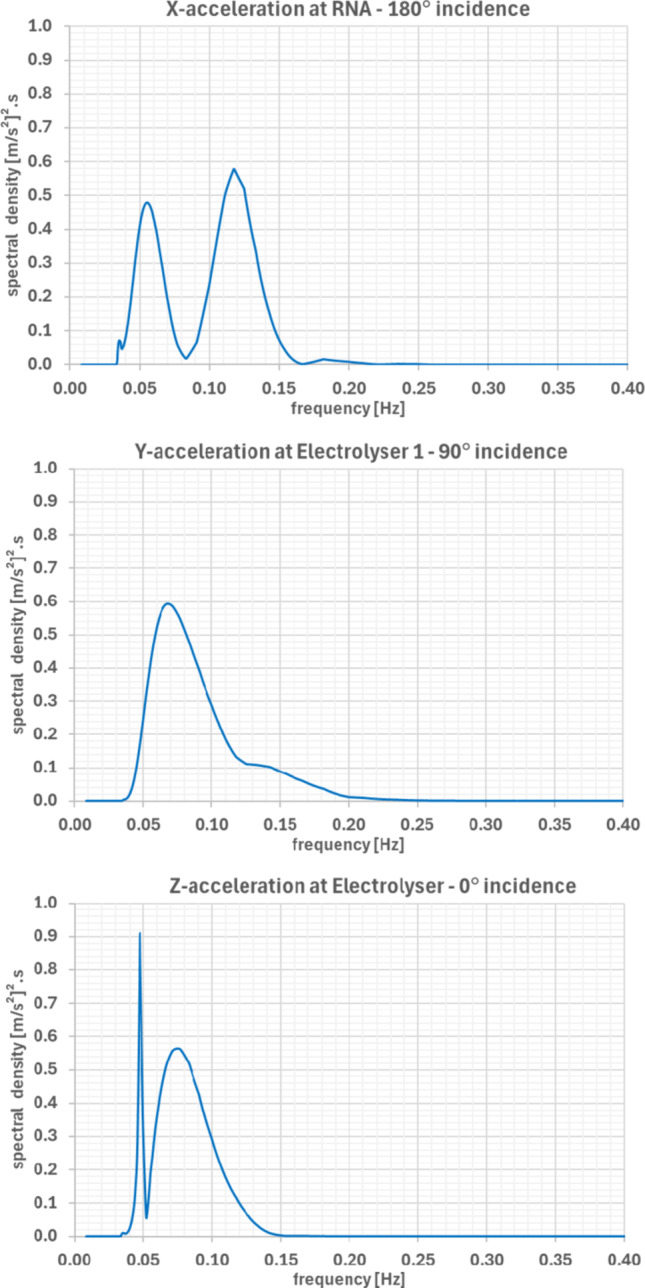
Response spectra of the most critical acceleration components due to survival sea state at the Ica region

Response spectra for the most critical accelerations under survival conditions (Fig. 12) show primary responses near the wave peak frequency (~ 0.06 Hz) but also secondary peaks linked to platform natural frequencies—e.g., x-acceleration coupling with pitch (~ 0.08–0.15 Hz) and z-acceleration peaks linked to heave resonance (~ 0.050 Hz).

Pitch motions, critical for turbine performance, remain small: maximum significant amplitudes of ~ 0.6° (survival) and 0.2° (operational) at 180° incidence, far below DNV-RP-0286 thresholds (5° mean, 10° max). For reference, the static pitch from maximum operational thrust and drag is ~ 9°, suggesting that additional wind-induced contributions should be considered in full load case time-domain simulations.

**Fig. 13:**
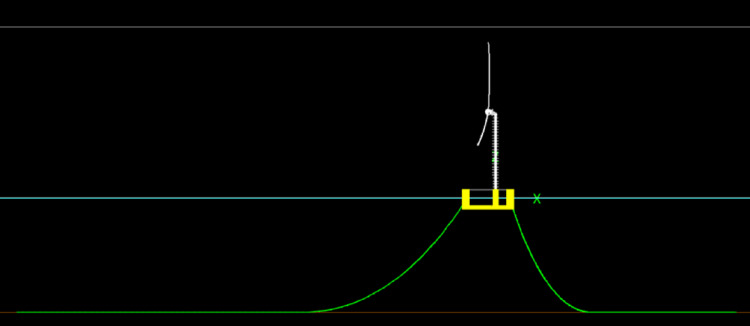
Time domain coupled aero-hydro-elastic-servo-control model of the FWHS in OrcaFlex®

**Time-domain.** Since the FWHS integrates a floating wind turbine, a time-domain approach is essential to capture the coupled aero-hydro-elastic-servo dynamics that cannot be fully represented in the frequency domain. In particular, wind loads, turbine control actions, mooring dynamics, and structural elasticity introduce nonlinearities that strongly influence platform motions and system responses. The FWHS was modelled in OrcaFlex® [17], with turbulent wind fields generated using TurbSim [18] in accordance with IEC 61400-1 [19] guidelines. Three representative Design Load Cases (DLCs) were considered, among which DLC 1.6 (rated operational condition under severe seas) is presented here as a reference scenario. Table 5 presents the representative design load cases (DLCs) adopted for the Ica region while Fig. 13 shows the OrcaFlex model of the integrated system.

**Fig. 14:**
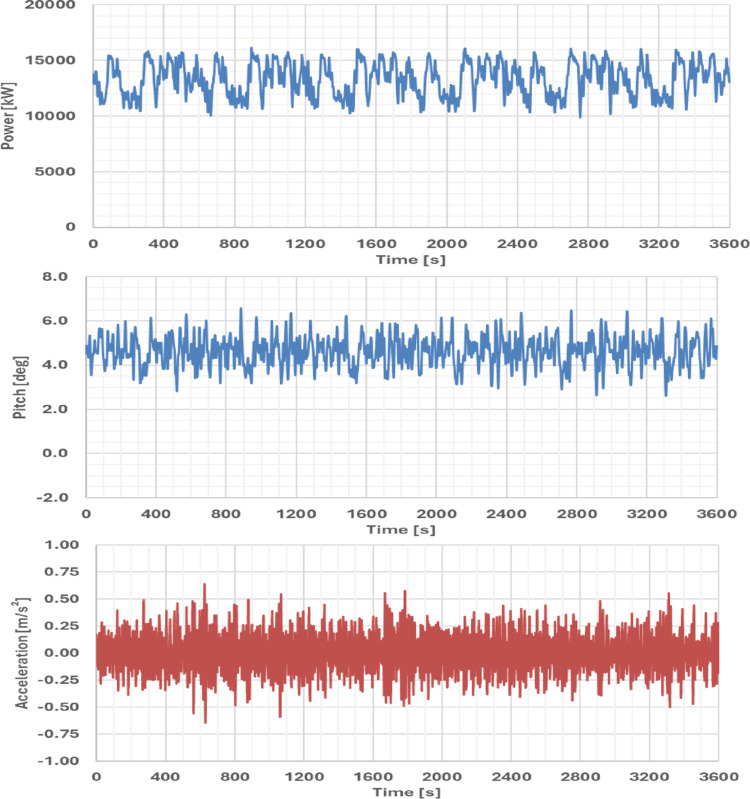
Time series results: **a** WT generated power, **b** platform’s pitch, **c** z-component acceleration at electrolyser 1 location for DLC 1.6 (0° colinear wave & wind)

The DLC 1.6 case was defined using a 50-year return wave condition with a collinear wind field at rated speed (~ 10.5 m/s). Results show that the maximum pitch angle did not exceed 7°, satisfying the DNV-RP-0286 criteria for floating wind turbines. This is a critical scenario since turbine thrust is maximized, yet the platform response remains within allowable limits. In contrast, under survival conditions with higher mean wind speeds but a parked turbine, pitch excursions are substantially lower due to the reduced thrust contribution. Acceleration levels at the RNA and electrolyser locations are in close agreement with frequency-domain predictions, though slightly reduced due to the inclusion of nonlinear damping effects, here modelled following values reported for the Volturn-US IEA 15 MW semisubmersible FOWT [10]. Importantly, both pitch and acceleration responses are lower than those obtained for the NE8 benchmark site, highlighting the favourable dynamic environment at Ica. Figure 14 presents typical simulation results for generated turbine power, pitch response and accelerations at electrolyser 1 location. The wind turbine power output in the simulations was predominantly governed by the wind field; however, wave-frequency effects were still visible as small modulations in the generated power. Based on the one-hour simulation time series and assuming a specific energy consumption of 56 kWh/kgH₂ for PEM electrolysis, the estimated hydrogen production for DLC 1.6 is ~ 191 kg/h. Although this value represents a short-term scenario under prescribed wind and wave conditions, it provides a useful validation of the modelling approach and highlights the potential for coupling time-domain simulations with hydrogen yield estimation models.

### Hydrogen production potential

Beyond short-term simulations, long-term hydrogen production potential was estimated from the 20-year hindcast metocean dataset for the Ica site. The analysis combined the IEA 15 MW wind turbine power curve with electrolyser constraints, assuming a single 12.1 MW PEM electrolyser operating with a minimum load threshold of 5% and constant efficiency (56 kWh/kgH₂). Hourly wind speeds were used to compute the incident power input, while downtime periods were defined as hours when turbine output fell below the minimum electrolyser load.

Under these assumptions, the annual hydrogen yield at Ica is estimated at ~ 1227 t/year, approximately 15% higher than that obtained for the NE8 site. The electrolyser utilisation factor reaches 64.5%, with only 11.4% of the generated wind energy curtailed, corresponding to an absorption efficiency of 88.6%. The system operates at its rated hydrogen production capacity during 44% of the year, reflecting the consistency of the wind regime in the region.

Downtime analysis indicates an average of 38 events per year, with a total duration of ~ 259 h/year. The maximum single downtime period in the 20-year record reached 80 h, but the median and 75th percentile values were 4 h and 8 h, respectively. In contrast, operational windows are notably long, with more than half exceeding 24 h and 75% lasting at least 9 h. These figures suggest that hydrogen production at Ica is not only reliable but also less fragmented than at NE8, reducing operational challenges.

**Fig. 15:**
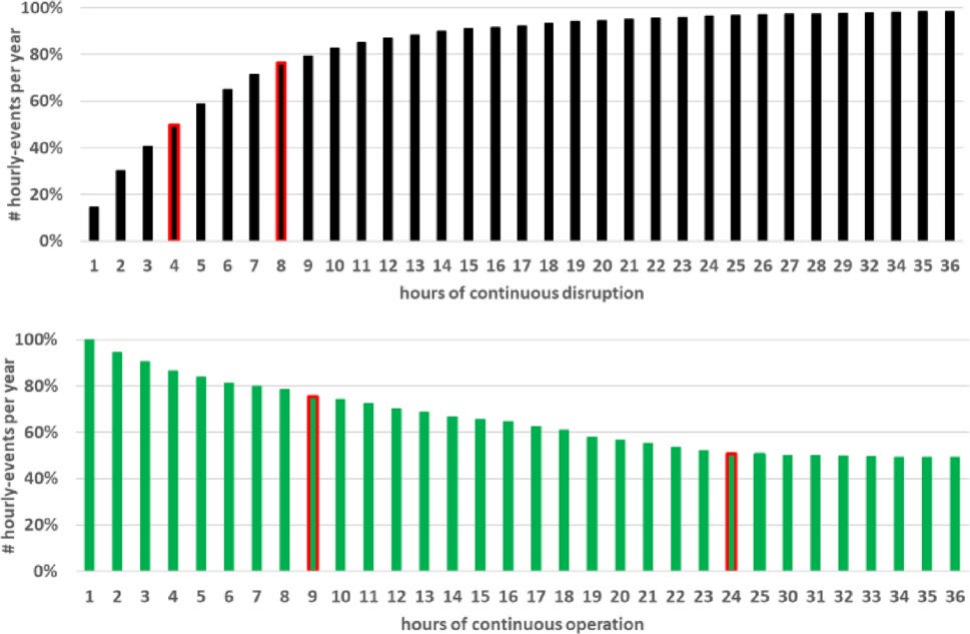
**a** Cumulative distribution of windows duration for H_2_ production based on 12-MW PEM electrolyser (5% of minimum load) and the 15-MW RWT at Ica Region: **a** downtime, **b** operational

Overall, the integration of the 15 MW FOWT with a 12 MW PEM electrolyser at Ica demonstrates robust performance, achieving high absorption of wind energy and limited downtime. Future work could explore modular electrolyser configurations to further reduce curtailment, though the stability of the Ica wind resource suggests this effect may be marginal. Figure 15 illustrates the cumulative distributions of downtime and operational windows derived from the long-term dataset.

## Conclusions

This study has presented the adaptation of a floating offshore wind–hydrogen system (FWHS), originally developed for a North Sea environment, to the offshore region of Ica, Peru. Using two decades of ERA5 hindcast data, we characterized the local wind and wave climate and assessed both operational and extreme conditions through frequency- and time-domain analyses. The main findings can be summarised as follows:

- **Wind resource and hydrogen potential.** The Ica offshore region combines high mean wind speeds with low variability, yielding a capacity factor of nearly 67% for the IEA 15-MW reference turbine. The long-term hydrogen yield is estimated at ~ 1227 t/year for a single 12-MW PEM electrolyser, exceeding the performance at the NE8 benchmark site despite lower full-power occurrence.
- **Favourable metocean environment.** Compared with Scottish waters, Ica exhibits milder extremes: 50-year return values of 14.9 m/s wind speed (10 m) and 4.9 m significant wave height, both substantially lower than NE8. This reduces curtailment risk, allows lighter substructures and mooring systems, and enhances the reliability of topside hydrogen equipment.
- **Hydrodynamic performance.** Frequency- and time-domain simulations confirm that accelerations and motions remain well below DNV design limits for both operational and survival states. Pitch excursions under DLC 1.6 remain < 7°, while electrolyser accelerations are an order of magnitude lower than allowable thresholds, indicating robust integration feasibility.
- **Infrastructure synergies.** The geographical proximity to major ports and the LNG terminal at Pampa Melchorita positions Ica as a strategic hub for hydrogen production, distribution, and potential export, aligning with global trends of repurposing LNG infrastructure for hydrogen and ammonia.

Overall, these results demonstrate that the Ica offshore region offers a uniquely favourable setting for deploying floating wind-hydrogen systems. Its stable wind regime, benign sea states, and emerging port infrastructure create conditions that are not only technically viable but also economically and strategically attractive.

Future work will refine the system design by incorporating coupled aero-hydro-servo-elastic simulations across the full IEC design load case matrix, assessing mooring and structural optimisations for Peruvian conditions, and exploring modular electrolyser layouts to further reduce curtailment. By doing so, this research can support Peru’s transition toward a green maritime economy and establish the Ica coast as a leading case study for offshore hydrogen production in emerging markets.

## Data Availability

No datasets were generated or analysed during the current study.
